# Mechanical Dentistry

**Published:** 1879-02

**Authors:** 


### BIBLIOGRAPHICAL.

*Mechanical Dentistry.* A Practical Treatise on the constitution of the various kinds of Artificial Dentures; comprising also useful formulæ, tables and receipts for gold plate, clasps, solders, etc. By Charles Hunter, Mechanical Dentist, London, Eng.—Publishers: Lindsay & Blakiston, Philadelphia, 1878.

While this work is perhaps the best, as it is the most recent treatise on Mechanical Dentistry originating from our English Colleagues, yet it is by no means so comprehensive and useful a text book as the part of Harris' Principles and Practice of Dentistry devoted to the same subject, and which was prepared by the late Prof. Philip H. Austen, who had few equals and no superiors in the demonstration and description of the manipulative methods of dental mechanism. This work of Mr. Hunter's consists of nineteen chapters devoted to Impressions and Plaster Models, Metal Models, Gold—Plate—Clasps—Wire, Soldering, Swaging Plates, Antagonizing Models, Mounting Teeth on Gold Plate, Vulcanite Work, Combination Work, Pivoting Teeth, Repairing Continuous Gum Work, Celluloid, Obturators and Artificial Palates, Manufacture of Vulcanite, and the Elastic Force of Steam, Metals used in Dentistry, Properties of Metals—Specific Gravity, Electro-Gilding, and Miscellanea, illustrated by 101 wood cuts.

Such subjects as relate to the preparation of the mouth preparatory to the insertion of artificial teeth, the selection and quality of materials employed in taking impressions, and the principles of the vacuum cavity, being essential to a treatise on Dental Mechanism, we are somewhat surprised that they do not appear in this work. It is also evident from other parts of this treatise, that our English Cousins are not quite up to the present status of the science as promulgated in this country; and while Mr. Hunter's may be regarded as an improvement on any similar treatise originating in Europe, yet it is by no means equal to some of our American text books. Notwithstanding such criticisms as we have made, many items may be gathered from this treatise which will prove interesting and instructive. The appearance of the work is very creditable to its well known American publishers.

